# DNA barcoding detects resurrected taxon *Giuris laglaizei* (Sauvage 1880) in Sulawesi, Indonesia: Bolano Sau Lake *payangka *phylogeny, phenotypic characters and implications for *Giuris *spp. conservation

**DOI:** 10.12688/f1000research.108970.2

**Published:** 2023-01-26

**Authors:** Samliok Ndobe, Muhammad Saleh Nurdin, Nur Hasanah, Aswad Eka Putra, Kasim Mansyur, Mohamad Nasir, Mashening L. Rabuna, Abigail Mary Moore

**Affiliations:** 1 Faculty of Animal Husbandry and Fisheries, Universitas Tadulako, Palu, Sulawesi Tengah, 94118, Indonesia; 2 Parigi Moutong District Marine and Fisheries Service, Petapa, Sulawesi Tengah, 94462, Indonesia; 3 Graduate School, Universitas Hasanuddin, Makassar, Sulawesi Selatan, 90245, Indonesia

**Keywords:** Eleotridae, Giuris margaritacea, amphidromy, phylogeny, meristic, morphometric, Tomini Bay, Wallacea

## Abstract

**Background**: The freshwater ichthyofauna of Wallacea is diverse and understudied. A baseline survey of Bolano Sau Lake in Parigi Moutong District, Central Sulawesi Province, Indonesia in 2019 found an eleotrid goby (local name payangka) with characters conforming to the genus *Giuris*, long considered monophyletic as *G. margaritacea/G. margaritaceus *but recently found to comprise at least eight species. This study focused on the molecular (DNA barcoding) identification and phenotypic characters of the payangka.

**Methods**: Payangka samples were collected from August to December 2019 in collaboration with local fishermen, weighed and measured, and preserved in 75% ethanol. Length, weight, sex (n=111) and 17 morphometric characters/six meristic counts (n=42) were recorded. DNA barcoding was performed on a fin clipping preserved in 96% ethanol. Homologous nucleotide sequences were obtained from public (GenBank and BOLD) databases, analysis conducted in MEGA X, and phylogenetic trees edited in the Interactive Tree of Life (iToL).

**Results**: Within the deeply divided *Giuris* clade, the payangka sequence resolved into a sub-clade identified as *Giuris laglaizei* (Sauvage 1880), a recently resurrected taxon, based on a sequence provided by Philippe Keith. The length-weight relationship (L = 0.0087∙W3.162) indicated mildly allometric positive growth. Size distribution differed significantly between male and female fish with significantly larger mean size of males (13.56 cm) than females (11.62 cm). The meristic formula was: D VI-I,8 A I,8 P 13 V I,5 C15. Phylogenetic analysis indicated four *Giuris* species in wetlands around Tomini Bay and five in Sulawesi.

**Conclusions**: This first record of *G. laglaizei* in Indonesia advances knowledge of Wallacean and Indo-Pacific Gobiiformes biogeography and highlights the need for a revision of the conservation status of the taxa currently grouped under *Giuris margaritacea/G. margaritaceus* in the IUCN Red List and FishBase databases. The data will inform biodiversity and fisheries management at local and regional levels.

## Background

Indonesian freshwater ichthyofauna is highly diverse, comprising primary freshwater and diadromous fishes. ^1^ While the ichthyofauna of western Indonesia (Sundaland biogeographic province) is dominated by cyprinids (Cyprinidae), the gobies (Gobiiformes) predominate in the Wallacea biogeographic province. ^1^^,^^2^ The freshwater ichthyofauna of Sulawesi, the largest island in Wallacea, is characterised by endemic freshwater species flocks and diadromous (mostly amphidromous *sensu* ^3^^,^^4^) taxa with marine larval stages. ^1^ These include gobies (Gobiiformes) of the families Gobiidae and Eleotridae, the latter also known as sleepers or gudgeons. ^1^^,^^5^^–^^9^ Although the number of diadromous (mostly amphidromous) gobies reported from the lakes, rivers and coastal waters around Sulawesi is increasing, with several recently described species and range extensions, ^10^^–^^18^ the ichthyofauna of most Sulawesian waterbodies is still largely unstudied. ^9^

Typically multi-species shoals of amphidromous goby postlarvae, with local names including nike, penja and duwo, are heavily fished in coastal waters around Sulawesi as they migrate to freshwater habitats, both as the main fish catch and as bycatch in anguillid glass eel fisheries. ^11^^,^^12^^,^^19^^–^^23^ Goby postlarvae are also present and intensively fished in some inland waters, in particular Tondano Lake in North Sulawesi. ^10^^,^^24^^–^^28^ The adults of some diadromous gobies are also locally important as food fish, including gudgeons (Eleotridae) generally known as payangka or (more rarely) payangga. ^10^^,^^29^^,^^30^ Sulawesian payangka populations have been identified as northern mud gudgeon *Ophiocara porocephala* (Valenciennes 1837) ^31^^–^^33^ and snakehead gudgeon *Giuris margaritacea* (Valenciennes 1837). ^25^^,^^26^^,^^34^^–^^36^ Both taxa are thought to be amphidromous. ^37^

In recent decades the Indonesian Government has promoted so-called “re-stocking” of inland waterbodies. These programs almost always involve the release of non-native (alien) species, often with negative impacts on native aquatic species in Indonesia ^38^^,^^39^ including in Sulawesi. ^40^^–^^43^ Increasing concern for native aquatic species has prompted surveys of inland waterbodies in Sulawesi, including those with a history of such introductions.

Bolano Sau Lake is one of a series of three small lakes close to the Tomini Bay coast of the northern arm of Sulawesi, in Bolano Subdistrict, Parigi Moutong District, Central Sulawesi Province. A baseline survey of Bolano Sau Lake in 2019 found an ichthyofauna dominated by introduced (alien) fish species. ^44^^,^^45^ Three native species were caught during sampling, one of which was a gudgeon with the local name payangka, tentatively identified as *Giuris margaritacea,* ^45^ the current valid name of the snakehead gudgeon in FishBase, the Global Database of Fishes. According to Kottelat (2013) the genus name *Giuris* is masculine in gender, and therefore the correct nomenclature is *Giuris margaritaceus*, the current organism name in the NCBI GenBank and BOLD nucleotide sequence databases. ^46^

Within the order Gobiiformes, cryptic and morphologically similar species can complicate identification based on external morphology, ^46^^–^^49^ and there have been many taxonomic revisions, including within the family Eleotridae. There are at least 10 “non valid” synonyms of *G. margaritacea* (Valenciennes 1837) (originally *Eleotris margaritacea* Valenciennes 1837) listed in FishBase, including *Ophieleotris aporos* (originally *Eleotris aporos* Bleeker 1854) and junior synonyms under the genera *Eleotris* (n=7), *Hypseleotris* (n=1), and *Ophieleotris* (n=1). *Ophieleotris aporos* in particular is still commonly used, ^14^^,^^30^^,^^50^^–^^52^ and a recent checklist of fishes from two Sulawesian islands ^13^ lists *G. margaritacea* (from East and West Timor) and *O. aporos* (from lakes in North Sulawesi) as separate species, with the further addition of *Ophieleotris* aff. *aporos* (from Buton Island in Southeast Sulawesi). Recent research has demonstrated that *G. margaritacea* has been erroneously and confusingly applied to a taxonomic group comprising at least eight species. ^15^^,^^53^

Such taxonomic uncertainty seriously complicates accurate species determination and the search for valid information on aspects such as species distribution, biology, ecology and status. As pointed out by, ^54^ the ability to precisely identify the fish species in fisheries catches or waters is important for moving towards more sustainable exploitation of fish resources and better protection of fish diversity. An increasingly common molecular approach to species identification is DNA barcoding; this involves the sequencing of a fragment of DNA which is highly conserved within species but differs between species of the taxonomic group being studied. ^55^ For vertebrates, including fishes, a subset or fragment of the mitochondrial cytochrome oxidase I (COI) gene is the most commonly used barcoding region. ^2^^,^^56^ In addition to species identification, phenotypic traits are important for taxonomy and to support fisheries management. ^54^^,^^57^^,^^58^

From a biodiversity and responsible fisheries management point of view, it was considered important to determine the native species currently present in Bolano Sau Lake. This study combined molecular biology methods (DNA barcoding) to identify the payangka in Bolano Sau Lake with classic methods to describe phenotypic characters, in particular external morphology (morphometric and meristic characters) and growth pattern (length-weight relationship). The study will inform management of the payangka as well as contributing to knowledge of *Giuris* biogeography *.*

## Methods

### Ethical statement

This study complied with relevant ethical regulations in Indonesia and followed the ARRIVE guidelines. The use of the samples in this study did not require specific ethical approval for the following reasons:

1. All fish specimens used were obtained under an ongoing collaboration between the Parigi Moutong District Marine and Fisheries Service (Dinas Kelautan dan Perikanan Kabupaten Parigi Moutong) and Universitas Tadulako following all applicable regulations.
2. The fish were captured by local fishers operating legal fisheries using permitted artisanal fishing gears.
3. All fish were euthanized following the standard guidelines in use for fish specimens at Universitas Tadulako. Based on standard internationally recognised protocols, ^59^ the procedures used were designed to minimise any suffering experienced by the specimens (through anaesthetising the fish before pithing), and were performed by experienced personnel.
4. The IUCN Red List assessment lists *Giuris margaritacea* (the only listed *Giuris* taxon) in the Least Concern category (not considered at risk of extinction).
5. There was no experimental component.

### Study site

**Figure 1.**
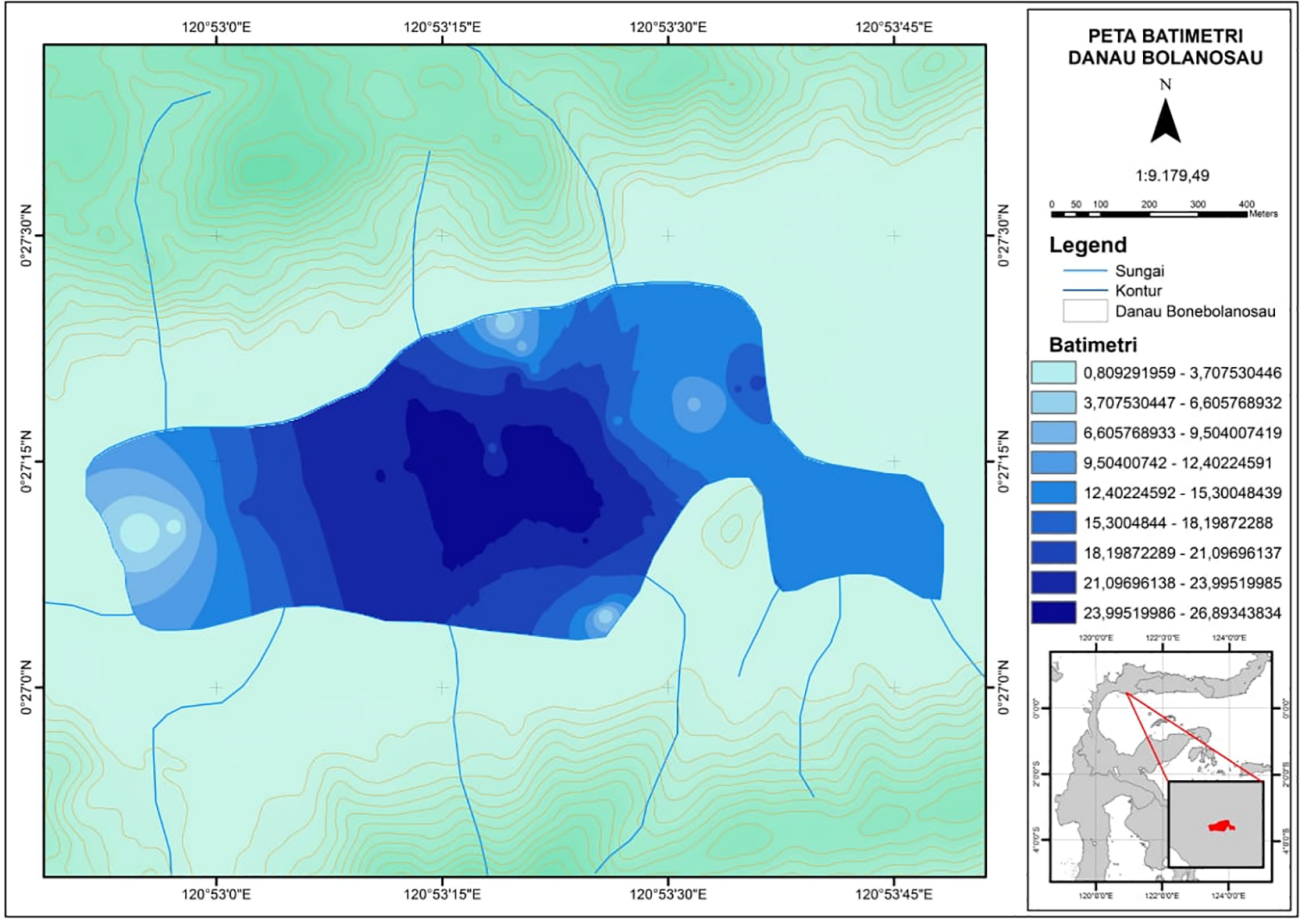
Bathymetric map of Bolano Sau Lake in Parigi Moutong District, Central Sulawesi, Indonesia.

Bolano Sau is the largest of a three-lake complex in the coastal plain along the north coast of Tomini Bay in Parigi Moutong District, Central Sulawesi Province, Indonesia. Batudako Lake is further inland, and Laut Kecil Lake is closer to the coast. Bolano Sau Lake is situated just north of the equator in Bolano Barat Village, Bolano Subdistrict, between approximately 0° 27’ 12” to 0° 27’ 15” N and 120° 52’ 52” to 120° 53’ 49” E an elevation of 5 m above sea level with an area of around 76 Ha, an average depth of around 4.26 m and a maximum depth of less than 10 m ( Figure 1).

### Fish specimens

Payangka specimens for morphometric and meristic analysis were collected from August to December 2019 in collaboration with local fishermen using a throw net with mesh size 3.5” (n=42). Field identification followed. ^6^^,^^60^ Specimens were humanely euthanized (anaesthesia with 70% ethanol followed by pithing) following. ^59^ Each fish was then weighed (electronic scales, precision 1 g), measured (Cadwell fish ruler, precision 0.5 mm), labelled and preserved in 70% alcohol.

### DNA extraction and barcoding

Prior to preservation, a fin clipping was taken from the right-hand pectoral fin of a *payangka* specimen (female, total length (TL)=13.32 mm) and placed in a 1.5 mL Eppendorf tube filled with 96% absolute ethanol. The sample was dispatched to the BIONESIA laboratory in Denpasar for DNA barcoding. Genomic DNA was extracted from the sample using the Qiagen DNeasy Blood & Tissue Kit following manufacturer’s protocols. A fragment of mitochondrial DNA (mtDNA) from the cytochrome oxidase I (COI) gene was amplified through polymerase chain reaction (PCR) using Fish_F1 and Fish_R1 primers. ^61^ The PCR profile comprised initial denaturation at 94 °C for 3 min; 35 cycles of 94 °C for 30 s, 50 °C for 30 s, and 72 °C for 60 s; and final extension at 72 °C for 2 min. The PCR product was visualized *via* electrophoresis on 1% agarose gel stained with Nucleic Acid Gel Stain (GelRed ^®^). Sanger sequencing of the PCR product was performed by First Base (Singapore). The forward and reverse sequences were trimmed, aligned and combined in MEGA X ^62^ to produce a nucleotide sequence submitted to the NCBI GenBank repository as accession OM674613. ^63^ The nucleotide composition (adenine, thymine, guanine, cytosine bases) of the *payangka* mitochondrial cytochrome C oxidase subunit I (COI) gene sequence was determined in MEGA X. ^62^ The online National Center for Biotechnology Information (NCBI) standard nucleotide Basic Local Alignment Search Tool-nucleotide (BLAST ^©^) blastn routine and the Barcode of Life Database (BOLD) Identification routine were used to provide an initial identification.

### Phylogenetic analysis

Homologous nucleotide sequences with at least 90% coverage were obtained from the NCBI blastn results (Gobiiformes in the 100 closest matches) and NCBI GenBank accession search using the terms *Giuris margaritaceus*, *G. margaritacea*, *Ophieleotris aporos* and *Ophiocara porocephala*, as well as from the BOLD Database using the keyword *Giuris* which yielded seven Barcode Index Numbers (BINs), with additional sequences obtained from the scientific literature (Table S1 – see Extended Data). The climbing perch *Anabas testudineus* was used as an outgroup: a sequence from Bolano Sau Lake, GenBank accession OM674614, ^63^ and GenBank accession MG407353. ^64^ All alignment, trimming and evolutionary analyses were conducted in MEGA X. ^62^ Evolutionary relationships were inferred and phylogenetic trees constructed using the Maximum Likelihood method and Kimura 2-parameter model ^65^ with default parameters, all codon positions, 100 × bootstrap test, and branch lengths representing the number of substitutions per site.

The first phylogenetic tree was constructed from an aligned dataset with 630 nucleotide positions containing 96 nucleotide sequences (GenBank accessions in Table S1). A second tree was constructed using all sequences in Table S1 with the genus level label *Giuris* (including BOLD records and non-deposited sequences) and other sequences nested within the *Giuris* clade in the first tree; there were 94 nucleotide sequences with 580 nucleotide positions in the aligned dataset. The payangka sequence was included in both analyses. The phylogenetic trees were exported from Mega X as Newick tree files and edited in the on-line interactive Tree of Life (iToL). ^66^^,^^67^ Pairwise evolutionary distances (number of base substitutions per site) within the *Giuris* and an outgroup ( *Mogurnda adspersa*) from the nearest Eleotridae clade were estimated using the Compute Pairwise Distances routine in Mega X, using the Maximum Composite Likelihood model. ^68^

### Phenotypic characteristics of the Bolano Sau Lake payangka

**Figure 2.**
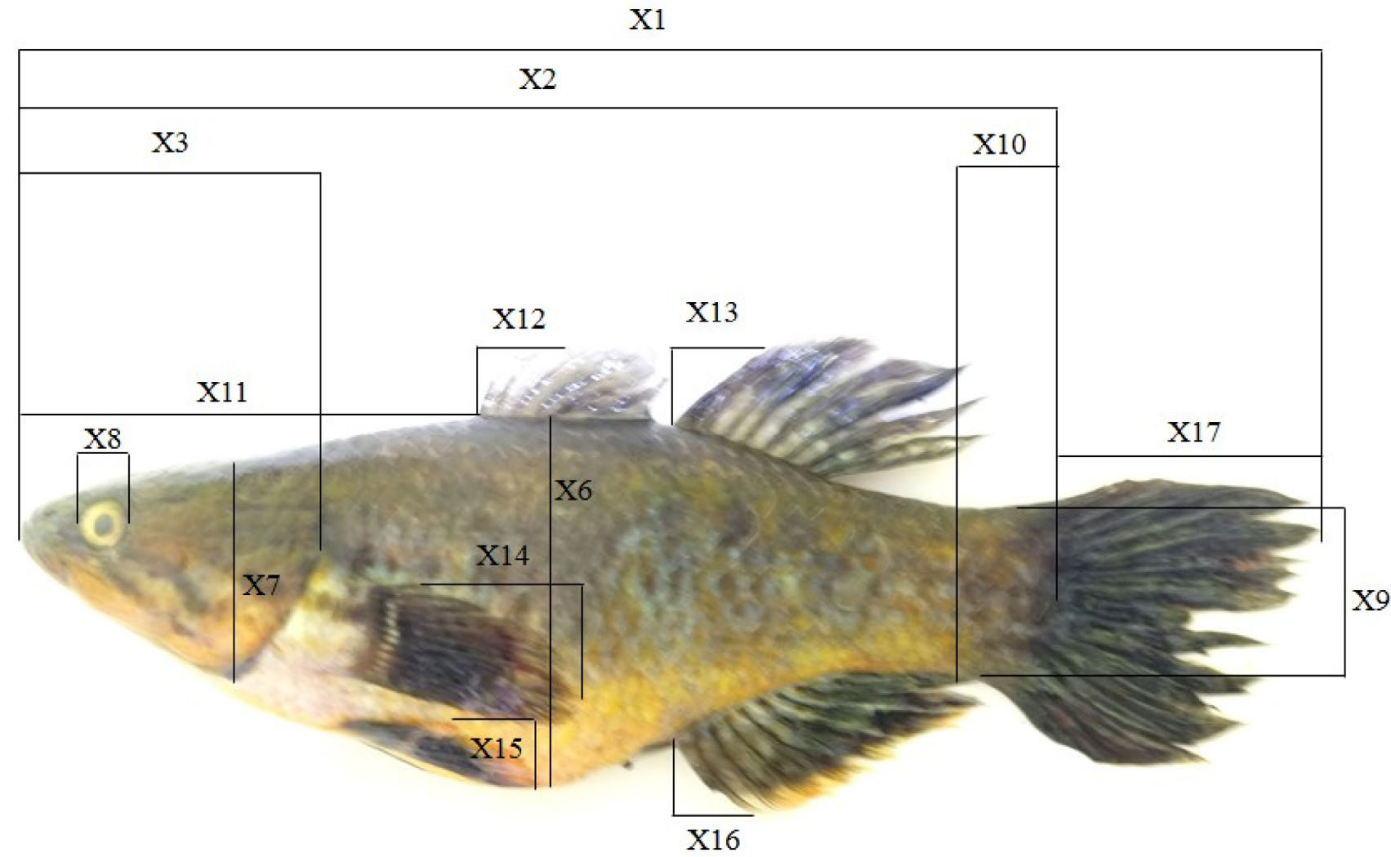
Morphometric characters of Bolano Sau Lake payangka measured in this study.

Morphometric and meristic characters ( Table 1) of the Bolano Sau Lake payangka (n=42) were measured or counted in the Aquatic Biology Laboratory, Universitas Tadulako, Palu. ^69^ Morphometric characters ( Figure 2) were measured using electronic callipers with a precision of 0.01 mm. Length, weight, sex and gonad maturity status data from a study on payangka reproductive biology (n=69; 25 females and 44 males) collected from Bolano Sau Lake in August and October 2019 ^45^ were also included in some analyses. Data were tabulated in Microsoft Excel 2010 and analysed descriptively. The length-weight relation analysis (n=107) was performed in Microsoft Excel 2010 (RRID:SCR_016137) using the log _10_-transformed version of the formula W= *a∙*L ^b^, where W is total body weight (g); L is total length (cm); *a* is the antilog of the intercept and *b* is the slope of the linear regression of the Log _10_ transformed data. Analysis of mean size and size class distribution were implemented in R version 3.6.0 (RRID:SCR_001905) ^70^ through the Rstudio version 1.1.456 interface (RRID:SCR_000432), ^71^ using code from ^72^ with a size class interval of 1 cm. Microsoft Excel spreadsheet algorithms based on ^72^ were used to estimate mean size at first maturity (L50) and sex-ratio by size-class, also with a size class interval of 1 cm. The meristic formula was based on median values of the 6 meristic characters (dorsal fins D1 and D2, anal fin A, pectoral fin P, ventral fin V, caudal fin C) with spine counts given in Roman numerals and ray counts in Arabic numerals. Selected characters were compared with data on other *Giuris* spp. populations.

**Table 1.** Morphometric and meristic characters of payangka used in this study.

Morphometric characters (see Figure 2)				Meristic counts	
Code	Description	Code	Description	Code	Description
X1-TL	Total length	X10-LP	Length of caudal peduncle	C	Caudal fin rays
X2-SL	Standard length	X11-SD	Tip of snout to base of anterior dorsal fin	A	Anal fin spines/rays
X3-HL	Head length from tip of snout to operculum margin	X12-DA	Length of first anterior dorsal fin spine	D1	Anterior dorsal fin spines/rays
X4-UJ	Upper jaw length	X13-DP	Length of first posterior dorsal fin spine	D2	Posterior dorsal fin spines/rays
X5-LJ	Lower jaw length	X14-PF	Length of pectoral fin	P	Pectoral fin rays
X6-BD	Body depth (maximum)	X15-VF	Length of ventral fin	V	Ventral fin rays
X7-HH	Head height	X16-AF	Length of first anal fin spine		
X8-ED	Eye diameter				
X9-CP	Height of caudal peduncle	X17-CL	Tail (caudal) length		

### Biodiversity conservation and fisheries

Qualitative data on biodiversity and fisheries in Bolano Sau Lake were collected during the payangka sample and environmental data collection. Secondary data were sourced from the baseline survey carried out from August to October 2019, ^44^ scientific literature, and reputable on-line sources. Primary and secondary data were analysed with respect to implications for biodiversity, including taxon conservation status, and fisheries management.

## Results

### Species identification and phylogenetic analysis

**Figure 3.**
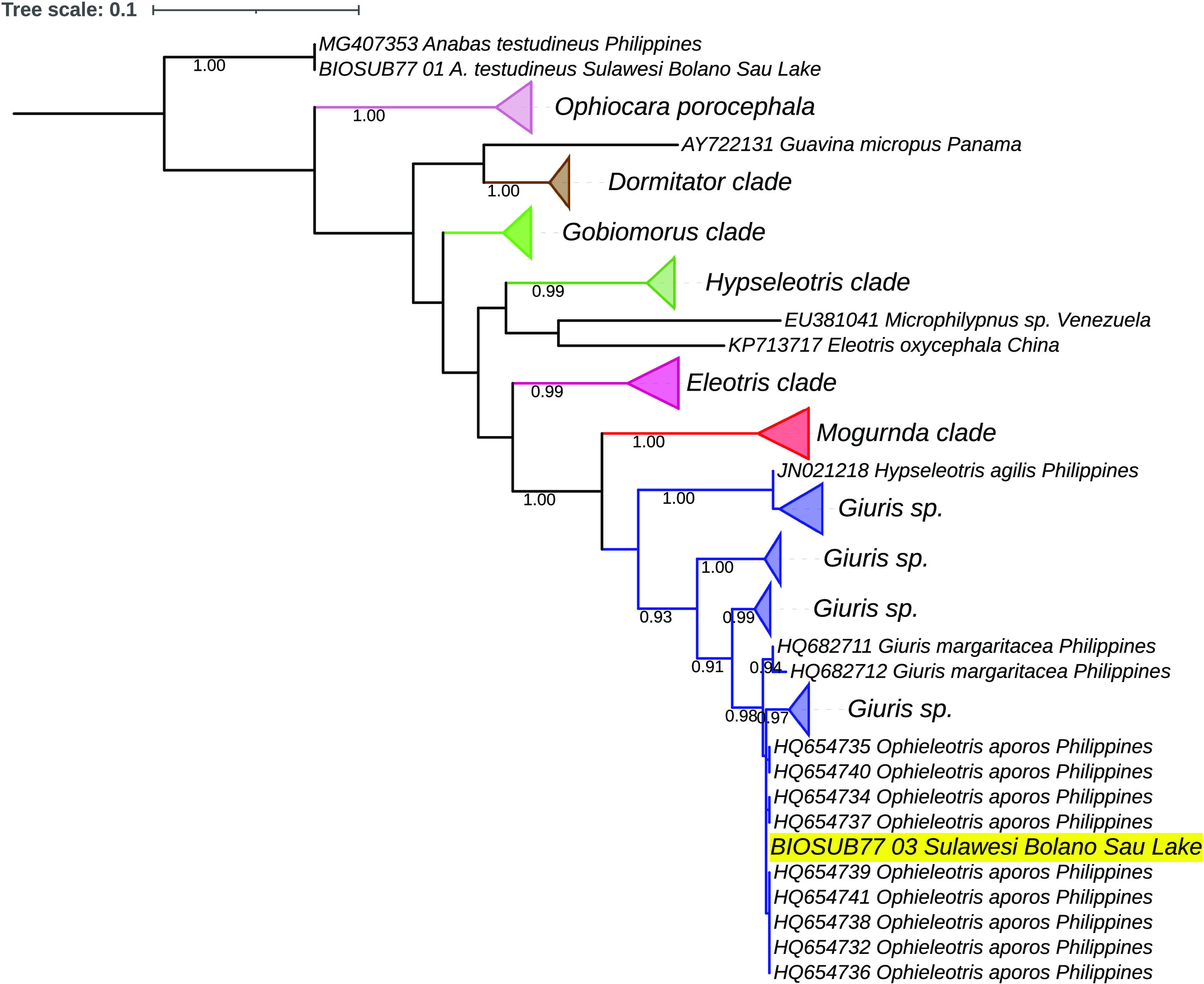
Evolutionary relationships of payangka from Bolano Sau Lake with several gobioid taxa and *Anabas testudineus* as an outgroup. The Bolano Sau Lake payangka sequence is highlighted in yellow. Evolutionary relationships were estimated using the Maximum Likelihood routine in MEGA X. ^62^ The analysis comprised 96 sequences with 630 nucleotide positions and 100 bootstrap replicates. The blue clade comprises sequences deposited in GenBank or BOLD databases as *Giuris margaritacea* and synonyms including *Ophieleotris aporos.* The length of the triangles is proportional to the number of sequences. The branch scale is in number of substitutions per site.

The nucleotide composition (nitrogen bases) of the mitochondrial cytochrome C oxidase subunit I (COI) gene sequence was guanine 19.3%, cytosine 29.0%, adenine 23.9% and thymine 27.8%. The NCBI blastn and BOLD Identification routines assigned the goby or gudgeon, known locally as payangka, to the Gobiiformes, Family Eleotridae, genus *Giuris* and taxonomic group labelled as *Giuris margaritacea.* Ten snakehead gudgeon *Giuris margaritaceus* sequences from Taal Lake, Luzon in the Philippines (accessions HQ654732-HQ654740) originally deposited as *Ophieleotris aporos* ^52^ had a very high similarity (99.84%–99.85%) with the payangka sequence from Central Sulawesi, Indonesia, GenBank accession OM674613. The condensed tree in Figure 3 shows the *payangka* sequence (labelled BIOSUB77_03) nested within a *G. margaritaceus* sub-clade containing these sequences. Additional analyses using the Neighbor-Join option in MEGA X produced an equivalent structure. Seven other Philippine sequences had similarities of 98.45–99.38%, including accessions HQ682711 and HQ682712 from Laguna Lake, also in Luzon ^73^ and accessions MG407388 to MG407392 from Lanao Lake in Mindanao. ^64^

**Figure 4.**
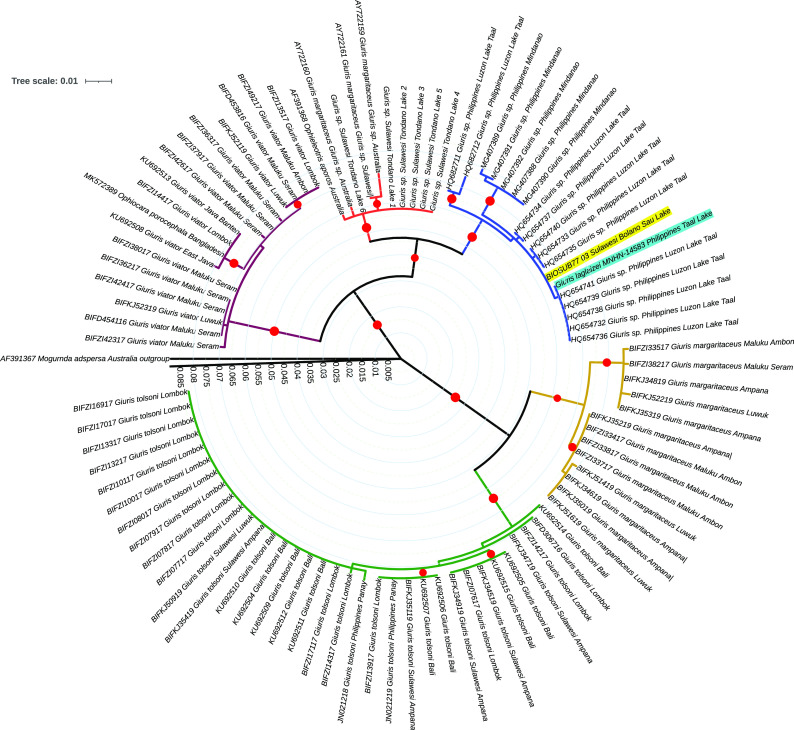
Evolutionary relationships of the genus *Giuris*, with *Mogurnda adspersa* as outgroup. The Bolano Sau Lake payangka sequence is highlighted in yellow and the *Giuris laglaizei* sequence MNHN-14583 from Philippe Keith is highlighted in turquoise. The Maximum Likelihood analysis in MEGA X ^62^ included 94 sequences with 580 positions and 100 bootstrap replicates. The red circles indicate node bootstrap values over 75%. The branch scale is in number of substitutions per site. Accession/Record details: see Table S1.

The analysis of the *Giuris* clade incorporating sequences from ^15^ retrieved from the BOLD database ( Figure 4) shows that the payangka from Bolano Sau Lake is not closely related to other *Giuris* specimens from Sulawesi or other regions in Indonesia. The number of base differences per site for representative sequences from each clade in Figure 4 ( Table 2) shows that the genetic distance between the payangka and *Giuris* sp. from the Philippines was 0.002 to 0.016. Meanwhile the genetic distance between payangka and sequences from Indonesia in other *Giuris* clades was between 0.064 and 0.126, a range consonant with congeneric rather than conspecific relationships.

**Table 2.** Genetic distances between payangka and selected *Giuris* sp. from Philippines, Indonesia and Australia (part 1).

No	Sequence details			Sequence number										
	Accession/Record	Taxon ^a^	Origin ^b^	1	2	3	4	5	6	7	8	9	10	11
1	BIOSUB77_03	GS1	CS-BS											
2	HQ654740	GS1	PH-L	0.002										
3	HQ654739	GS1	PH-L	0.002	0.003									
4	HQ682712	GS1	PH-L	0.013	0.014	0.014								
5	MG407390	GS1	PH-M	0.013	0.014	0.014	0.026							
6	MG407389	GS1	PH-M	0.016	0.018	0.018	0.029	0.003						
7	None	GS2	NS-T	0.029	0.031	0.031	0.039	0.032	0.032					
8	AY722161	GS2	SUL	0.034	0.036	0.036	0.044	0.037	0.037	0.005				
9	AF391368	GS2	AUS	0.031	0.032	0.032	0.041	0.034	0.034	0.002	0.006			
10	BIFKJ35219	GM	CS-A	0.122	0.124	0.124	0.135	0.120	0.122	0.120	0.126	0.122		
11	BIFKJ51419	GM	CS-L	0.126	0.128	0.128	0.139	0.124	0.126	0.120	0.126	0.122	0.003	
12	BIFZI33517	GM	MAL	0.111	0.112	0.112	0.123	0.109	0.111	0.109	0.115	0.111	0.018	0.021
13	BIFKJ52319	GV	CS-L	0.066	0.066	0.068	0.077	0.077	0.077	0.059	0.064	0.061	0.130	0.126
14	BIFZI36317	GV	MAL	0.068	0.069	0.069	0.079	0.078	0.078	0.061	0.066	0.062	0.124	0.120
15	BIFZI13517	GV	LO	0.066	0.068	0.068	0.077	0.076	0.076	0.062	0.068	0.064	0.126	0.122
16	KU692513	GV	J-B	0.064	0.066	0.066	0.075	0.075	0.075	0.061	0.066	0.062	0.124	0.120
17	KU692508	GV	J-E	0.066	0.068	0.068	0.077	0.076	0.076	0.062	0.068	0.064	0.126	0.122
18	BIFKJ34919	GT	CS-A	0.121	0.119	0.122	0.133	0.121	0.123	0.115	0.121	0.117	0.051	0.054
19	BIFKJ50919	GT	CS-L	0.119	0.117	0.121	0.131	0.119	0.121	0.113	0.119	0.115	0.054	0.058
20	BIFZI08017	GT	LO	0.119	0.117	0.121	0.131	0.119	0.121	0.113	0.119	0.115	0.054	0.058
21	KU692506	GT	BAL	0.124	0.123	0.126	0.137	0.124	0.126	0.119	0.125	0.121	0.054	0.054
22	JN021219	GT	PH-P	0.124	0.123	0.126	0.137	0.124	0.126	0.119	0.125	0.121	0.054	0.054
23	AF391367	MA	AUS	0.145	0.147	0.147	0.158	0.147	0.143	0.141	0.145	0.143	0.161	0.163

^a^ Taxon: GS = *Giuris* sp.; GM = *Giuris margaritaceus*; GT = *Giuris tolsoni*; GV = *Giuris viator*; MA = *Mogurnda adspersa* (outgroup).

^b^ Origin: AUS = Australia; AUS/S = Australia or Sulawesi (actual site unknown); PH= Philippines; LU = Luzon (Lake Taal); M = Mindanao; P = Panay; Indonesia: CS= Central Sulawesi: A = Ampana; BS = Lake Bolano Sau; L = Luwuk; NS-T = North Sulawesi, Tondano Lake; SUL = Sulawesi, location unknown; BAL = Bali; LO = Lombok; MAL = Maluku; J-B = Java, Banten; J-E = East Java.

**Table 2.** (continued) Genetic distances between payangka and selected *Giuris* sp. from Philippines, Indonesia and Australia (part 2).

No	Sequence details			Sequence number										
	Accession/Record	Taxon ^a^	Origin ^b^	12	13	14	15	16	17	18	19	20	21	22
1	BIOSUB77_03	GS1	CS-BS											
2	HQ654740	GS1	PH-L											
3	HQ654739	GS1	PH-L											
4	HQ682712	GS1	PH-L											
5	MG407390	GS1	PH-M											
6	MG407389	GS1	PH-M											
7	None	GS2	NS-T											
8	AY722161	GS2	SUL											
9	AF391368	GS2	AUS											
10	BIFKJ35219	GM	CS-A											
11	BIFKJ51419	GM	CS-L											
12	BIFZI33517	GM	MAL											
13	BIFKJ52319	GV	CS-L	0.015										
14	BIFZI36317	GV	MAL	0.113	0.005									
15	BIFZI13517	GV	LO	0.115	0.006	0.005								
16	KU692513	GV	J-B	0.113	0.011	0.009	0.011							
17	KU692508	GV	J-E	0.115	0.013	0.011	0.013	0.002						
18	BIFKJ34919	GT	CS-A	0.061	0.133	0.130	0.129	0.127	0.125					
19	BIFKJ50919	GT	CS-L	0.065	0.131	0.128	0.127	0.125	0.123	0.006				
20	BIFZI08017	GT	LO	0.065	0.131	0.128	0.127	0.125	0.123	0.006	0.000			
21	KU692506	GT	BAL	0.065	0.129	0.127	0.125	0.123	0.121	0.006	0.006	0.006		
22	JN021219	GT	PH-P	0.065	0.129	0.127	0.125	0.123	0.121	0.006	0.006	0.006	0.000	
23	AF391367	MA	AUS	0.163	0.141	0.137	0.135	0.141	0.143	0.161	0.155	0.155	0.155	0.155

^a^ Taxon: GS = *Giuris* sp.; GM = *Giuris margaritaceus*; GT = *Giuris tolsoni*; GV = *Giuris viator*; MA = *Mogurnda adspersa* (outgroup).

^b^ Origin: AUS = Australia; AUS/S = Australia or Sulawesi (actual site unknown); PH= Philippines; LU = Luzon (Lake Taal); M = Mindanao; P = Panay; Indonesia: CS= Central Sulawesi: A = Ampana; BS = Lake Bolano Sau; L = Luwuk; NS-T = North Sulawesi, Tondano Lake; SUL = Sulawesi, location unknown; BAL = Bali; LO = Lombok; MAL = Maluku; J-B = Java, Banten; J-E = East Java.

### Phenotypic characters

**Figure 5.**
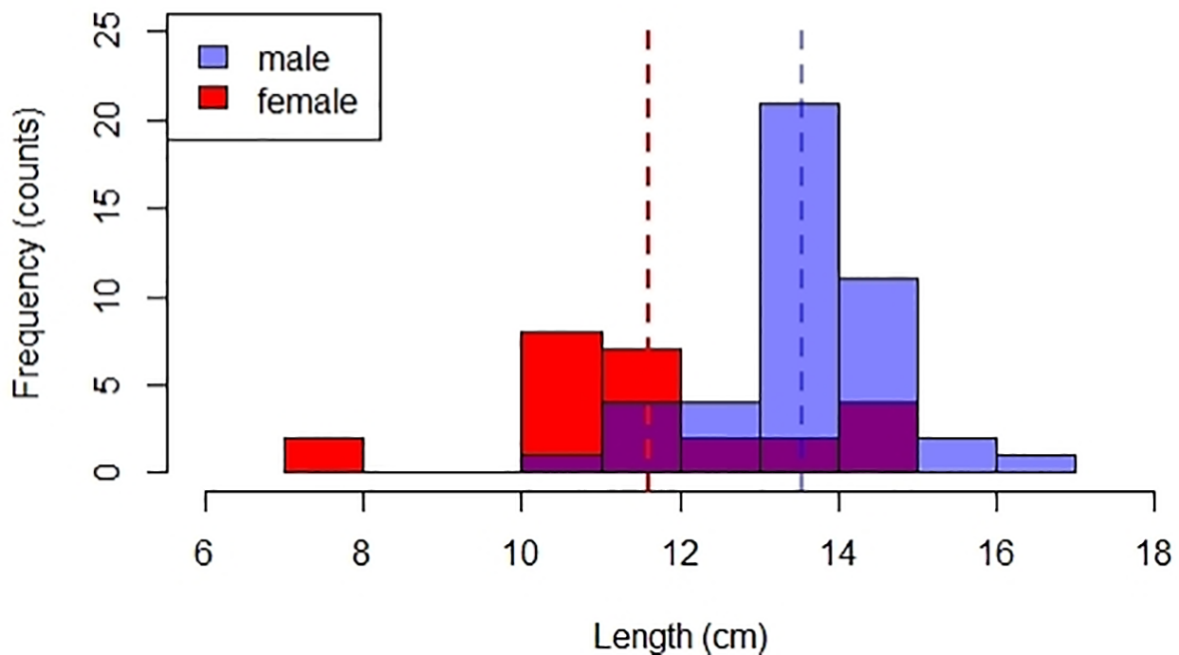
Total length (TL) distribution of male (n=44) and female (n=25) payangka ( *Giuris laglaizei*) from Bolano Sau Lake. Dotted lines indicate mean TL for male (blue) and female (red) fish.

The TL of *Giuris* sp. specimens (n=107) ranged from 7.9 cm to 16.3 cm, while weight ranged from 4.96 g to 61.0 g. The specimens in the morphometric and meristic study (n=42) ranged in size from 9.95 to 15.25 cm TL (mean 12.58 cm). In the sex-disaggregated length data set (n=69), variance was unequal between males and females (two-sample F-test, *P* < 0.01). The overall mean length was 12.95 cm. There was a highly significant ( *P* < 0.001, two-tail t-test assuming unequal variance) difference in mean length between males (13.56 cm TL, n=44) and females (11.62 cm TL, n=25), with overlapping length distributions ( Figure 5).

**Figure 6.**
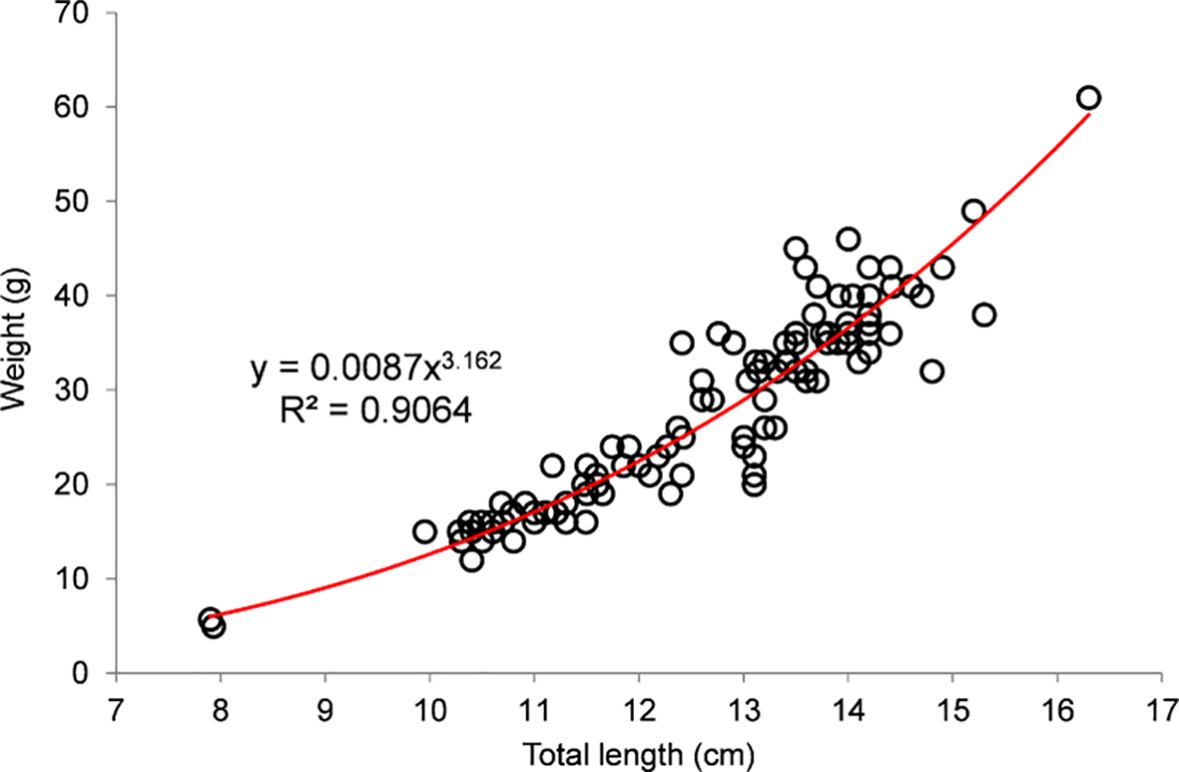
Length-weight relationship of payangka ( *Giuris laglaizei*) from Bolano Sau Lake (n=107).

Mean length at maturity (L _50_) was 9.3 cm TL for females and 11.5 TL for males. Sex ratio was significantly ( *P* < 0.05) different from 1:1 for all size classes except 12–13 cm TL, with female dominance below 12 cm TL and male dominance above 13 cm TL. The length-weight relationship was L=0.0087∙W ^3.162^ ( Figure 6), with a strong correlation (R ^2^=0.901) and *b* > 3, indicating a mildly allometric positive growth pattern.

A synopsis of the 17 morphometric characters measured for *Giuris laglaizei* from Bolano Sau Lake ( Table 3) presents the data as absolute values (in mm) and as dimensionless ratios to TL. These data indicate considerable variability in most characters. The 6 meristic counts of *Giuris laglaizei* from Bolano Sau Lake ( Table 4) yield a meristic formula based on median values of D VI-I,8 A I,8 P 13 V I,5 C15.

**Table 3.** Bolano Sau Lake payangka (n=42) morphometric characters.

Code ( Table 1)	Absolute values (mm)				Ratio to total length (X1-TL)			
	Minimum	Maximum	Mean	SD	Minimum	Maximum	Mean	SD
X1-TL	99.5	152.5	125.8	14.6	-	-	-	-
X2-SL	80.1	80.1	101.0	12.4	72.1%	90.0%	80.3%	2.8%
X3-HL	24.9	48.1	33.2	5.0	20.1%	31.5%	26.4%	2.0%
X4-UJ	6.0	9.1	7.3	0.8	4.1%	7.2%	5.8%	0.6%
X5-LJ	6.5	9.7	7.8	0.8	4.7%	7.8%	6.3%	0.6%
X6-BD	16.7	39.6	27.4	4.9	15.9%	27.4%	21.7%	2.1%
X7-HH	11.2	37.6	22.7	5.7	10.7%	27.0%	17.9%	3.3%
X8-ED	4.5	7.8	5.9	0.6	3.6%	6.0%	4.7%	0.5%
X9-CP	13.5	22.1	17.9	2.5	10.4%	16.5%	14.2%	1.0%
X10-LP	19.3	31.7	25.1	3.1	16.6%	26.7%	20.0%	1.9%
X11-SD	27.9	54.6	45.4	5.2	26.9%	39.4%	36.1%	2.2%
X12-DA	13.2	41.1	20.6	4.9	12.7%	27.0%	16.3%	2.9%
X13-DP	21.2	51.7	34.5	8.2	16.9%	36.0%	27.4%	5.4%
X14-PF	16.6	31.2	23.6	3.2	15.2%	21.4%	18.7%	1.3%
X15-VF	14.0	30.1	21.3	3.5	13.3%	20.4%	16.8%	1.5%
X16-AF	13.3	33.2	22.3	4.9	12.3%	22.4%	17.6%	2.7%
X17-CL	18.3	34.4	26.7	4.1	17.4%	25.7%	21.2%	1.7%

**Table 4.** Bolano Sau Lake payangka meristic characters (n=42).

Fin		Spines	Rays		
Code	Description		Minimum	Maximum	Median
D1	Anterior dorsal	VI *	-	-	-
D2	Posterior dorsal	I	7	8	8
A	Anal	I	8	10	9
P	Pectoral	-	12	15	13
V	Ventral	I	4	5	5
C	Caudal	0	11	17	15

* One specimen V.

### Environmental and fisheries data

**Figure 7.**
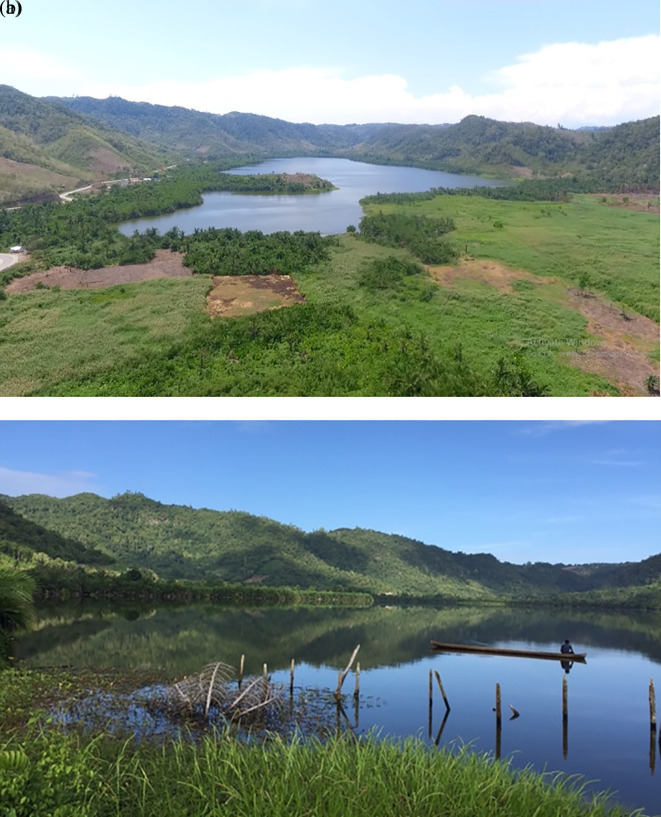
Photographs of Bolano Sau Lake in Central Sulawesi, Indonesia show extensive deforestation (a) and riparian wetlands with few remaining sago palms (b). (Photographs taken by Samliok Ndobe).

Water quality parameters tended to vary between sampling stations and times ( Table 5). The ranges considered normal for Indonesian freshwater bodies used for fisheries (classes 2 and 3) according to Government Regulation No. 82/2001 (RI 2001) are also provided for the parameters covered under this regulation. During the collection of payangka samples and water quality data, qualitative data on environmental conditions were noted. These included visual records (photographs) of general conditions; for excerpts from the visual record see Figure 7. Originally Bolano Sau Lake was surrounded by lowland tropical rainforest and sago palm dominated wetlands. Observations showed considerable anthropogenic impacts, including extensive land-use change leading to erosion and sedimentation. Extensive areas around the lake had been converted for agriculture and human habitation ( Figure 7a), and few sago palms remained in the riparian wetlands ( Figure 7b).

**Table 5.** Water quality data for Bolano Sau Lake (August-December 2019).

No	Parameter	Unit	Range	Standard ^109^
1	Water temperature	°C	31.30-33.42	Normal ± 3°C
2	Visibility	m	0.22-0.67	-
3	pH		8.06-8.57	6-9
4	Total suspended solids (TSS)	mg/L	75.04-183,99	-
5	Dissolved oxygen (DO)	mg/L	2.70-3.90	3-4
6	Total alkalinity	mg/L	1.5-11.5	-
7	Hardness	mg/L	174.28-222.54	-
8	Total ammonia nitrogen TAN (NH3+NH4 ^+^)	mg/L	0.05-0.07	-
9	Nitrate (NO3-N)	mg/L	<0.01-0.6	<0.02
10	Orthophosphate (PO4-P)	mg/L	<0.01	-

During the baseline survey, ^44^ fish samples collected through experimental fishing in collaboration with local fishermen using 3½″ gillnets. ^45^ Six species were reported: Nile tilapia *Oreochromis niloticus* (comprising nearly 77% of the experimental catch), payangka, striped snakehead ( *Channa striata*), climbing perch ( *Anabas testudineus*), gourami ( *Trichogaster* sp.), a tank goby ( *Glossogobius* sp.), and the Mozambique tilapia ( *Oreochromis mossambicus*). Local fishermen also reported catching common carp ( *Cyprinus carpio*) and freshwater eels ( *Anguilla* sp.) in the lake. This survey also revealed a predominantly sandy (67-73%) lake substrate with some silt (14-22%) and clay (11-13%). Phytoplankton concentration was 14,580/L, dominated by *Cylindrospermopsis* spp., *Dinobryon* spp., and *Cyclotella* spp. Mean zooplankton density was 28,890/L, dominated by unidentified larvae (Annelida, Crustacea (shrimps), Mollusca (bivalves) and fish) and *Paramecia.*

## Discussion

### *Giuris* phylogeny, biogeography and first species record

Barcode sequences (COI gene sequence fragments) over 600 bp are considered sufficient for differentiating between animal species, with 98-100% similarity generally indicating species identity. ^56^ The NCBI blastn and BOLD Identity routine results place the specimen collected (GenBank accession OM674614) within the taxon named as *Giuris margaritacea* (Chordata, Actinopterygii, Gobiiformes, Eleotridae) in these databases. Some unexpected sequence placements may have been due to errors in specimen identification, as might be expected within this taxonomic group. ^47^^,^^48^ Examples include the nesting of *Xenisthmus* sp. from Australia (accession AF391372 ^47^) in the *Mogurnda* clade, *Ophiocara porocephala* from Bangladesh (accession MK572389 ^74^) in a *Giuris margaritaceus* sub-clade, and well-defined clades with a mixture of species names in the genera *Eleotris* and *Gobiomorus.* The nesting of *O. porocephala* from Bangladesh in the *G. margaritacea* clade lends credence to the possibility that some reports of payangka as *O. porocephala* may be similar cases, and point to a possible history of mistaken identity between two taxa which are not closely related genetically.

First recorded and described from the Solomon Islands, ^75^ the snakehead gudgeon *G. margaritaceus* has been thought to have a widespread distribution in the Indo-Pacific region. ^76^ The gudgeon specimen examined by Valenciennes and named as *Giuris margaritacea* Valenciennes 1837, with the French common name *éléotris perlé*, was 6 inches long and collected from Vanikolo in the Solomon Islands by Quoy and Gaimard. ^75^ *G. margaritaceus* is reported from Madagascar ^77^ and the Indian sub-continent ^78^ to Papua New Guinea and Pacific Islands, ^75^^,^^77^^,^^79^^–^^82^ and from the Philippines ^52^^,^^83^ to northern Queensland and north-eastern Australia. ^47^^,^^77^^,^^84^^,^^85^ Considered native to Indonesia, it has been reported from Sumatra ^86^ in the west to Papua ^51^ in the east, including Sulawesi. ^6^^,^^14^

Historically, the large number of *G. margaritaceus* synonyms appears related to the geographical distribution of this taxon. Original references ( *e.g.* Cuvier and Valenciennes, 1837 ^75^ and Sauvage, 1880 ^87^) tend to give different species names to specimens collected from different islands or regions, even when they note a high level of similarity between morphological traits, with descriptions typically based on a small sample (in many cases just one specimen). Subsequent studies lead to a consensus view of the genus *Giuris* as monophyletic, with *G. margaritacea* or *G. margaritaceus* as the most senior (and hence valid) species name, as reflected in databases including the NCBI GenBank and BOLD, FishBase, the World Register of Marine Species (WoRMS), and Eschmeyer's Catalog of Fishes. However, Kottelat ^46^ noted that: “The wide distribution and the observed variability of *G. margaritaceus* suggests that more than one species might be confused under this name”. The structure of the trees in this study ( Figures 3 and 4) not only show deep divisions consonant with multiple species within *Giuris* but also indicate widespread misidentification within Eleotridae and a need for taxonomic revision within other eleotrid taxa.

The deep divisions within the putative *G. margaritaceus* clade in Figure 3 are similar to the deep divisions in *Glossogobius giuris* from India. ^49^ A study on the ichthyofauna of Java and Bali ^88^ reported two BOLD BINs for *Giuris margaritacea* from this region, with a genetic distance of 12.56%, indicating more than one species in this region of Indonesia. Recent studies on the genus *Giuris* within the Indonesian Archipelago ^15^ and other regions of the Indo-Pacific ^53^ collectively resurrect and redescribe three species previously synonymised with *G. margaritaceus* ( *G. laglaizei* Sauvage 1880; *Giuris aporocephalus* Macleay 1884; *Giuris tolsoni* Bleeker 1854), redescribe *Giuris margaritaceus*, and describe four new species ( *Giuris charpini* Keith & Mennesson 2020; *Giuris yahayai* Keith & Mennesson 2020; *Giuris caussei* Keith, Mennesson & Lord 2020; and *Giuris viator* Keith, Mennesson, Lord, Hubert 2020). However, the analyses in this study indicate the range distributions for *Giuris* species in Keith *et al.* (2020) and Keith & Mennesson (2020) ^15^^,^^53^ are still incomplete.

It is interesting that no *Giuris* sequence from Indonesia were closely related to the payangka from Bolano Sau Lake ( Figure 4), and evolutionary relationships within *Giuris* do not necessarily seem to follow readily discernible patterns based on past or present geographical distance. For example, as in Ref. 53, Australian and Philippine clades (the latter including payangka from Bolano Sao Lake) seem more closely related to each other than to species found in areas lying between these two regions ( Figure 4; Table 2), with one of the two barcoded lacustrine *Giuris* populations from the northern arm of Sulawesi (Bolano Sau and Tondano) belonging to each of these clades. The tree topography ( Figure 4) and location of the closest matching sequences (Taal Lake, Luzon, Philippines) strongly suggested that the payangka, as well as the Philippine sequences within which it is nested, are in fact *Giuris laglaizei* Sauvage 1880 (originally *Eleotris* (Giuris) *laglaizei*). This species was first described from a specimen with the local name poi-poi collected near Manila in the Philippines. ^87^ Unfortunately, sequences from Ref. 53 were not available in either GenBank or BOLD databases at the time of this study. However, Philippe Keith of the National Museum of Natural History of Paris kindly provided a sequence of *G. laglaizei* from the Philippines (Number 14583 in Ref. 53), which was not yet available in public databases. As expected, this sequence nested within the same *Giuris* clade as the payangka ( Figure 4). The identity was 99.83% (one nucleotide difference in the aligned dataset) with a genetic distance of ≈ 0.00136. Based on this result, the Bolano Sau Lake payangka can be identified as *G. laglaizei,* a first record for Sulawesi and Indonesia, considerably extending the known distribution of this species.

Comparison with Ref. 15 and Ref. 53 strongly suggests that the Australian sister clade to that containing the payangka in Figure 4 is most likely *Giuris aporocephalus* Macleay 1884. The six specimens from Tondano Lake resolved within this clade. If indeed the *Giuris* in Tondano Lake are descended from introduced payangka sourced from Limboto Lake over 100 years ago, then it is likely that the payangka in Limboto Lake also belong to this clade, and are therefore not the same species as the payangka in Bolano Sau Lake, even though these two lakes are relatively close to each other. However homologous barcode (mtDNA COI) sequences for Limboto Lake payangka are not yet available, despite recent research using the Cyt-b genetic marker. ^36^

Table 6 shows the known and (strongly) suspected species identities and distributions of species within the genus *Giuris.* This study brings the number of *Giuris* species in Indonesia and Sulawesi to five, with four species in freshwater ecosystems around the coasts of Tomini Bay.

**Table 6.** Species in the genus *Giuris* and their known (X) or suspected ( *X*) distributions.

No	*Giuris* Species ^a^			Indonesia ^b^							Other Countries ^c^						
		O	A	L	T	S	M	K	B	J	P	N	A	C	J	B	M
1	*G. aporocephalus*				***X***	***X***						X	X				
2	*G. charpini*													X			
3	*G. caussei*											X					
4	*G. laglaizei*	**X**									X						
5	*G. margaritaceus*		**X**	**X**			X				X	X		X			
6	*G. tolsoni*		**X**	**X**			X	X	X	X	X				X		
7	*G. viator*			**X**			X	X		X				X		X	
8	*G. yahayai*																X

^a^ Sources: No. 1-8: Ref. 53; No.1: inferred from Ref. 25 and Ref. 50; No. 4: this study; No. 5-7: Ref. 15; No. 7: Ref. 74.

^b^ O = Bolano Sau Lake (this study): A = Ampana; L = Luwuk; T = Tondano Lake, North Sulawesi (possibly also Limboto Lake in Gorontalo); S = Sulawesi, unknown site; M = Maluku; K = Lombok; B = Bali; J = Java; Grey shading and bold font = sites in Sulawesi.

^c^ P = Philippines; N = Papua New Guinea; A = Australia; C = Pacific islands; J = Taiwan and Japan (Okinawa); B = Bangladesh; M = Madagascar, Mayotte and Comoros.

### Morphometric and meristic characters within *Giuris*

Data on the length-weight relation of *Giuris* sp. appear to be limited and confined to Indonesia. Length-weight relation parameters and size ranges of payangka ( *Giuris laglaizei*) from Bolano Sau Lake and other *Giuris* populations in Indonesia ( Table 7) show a general tendency towards allometric positive growth patterns. The mildly allometric positive length-weight relation for Bolano Sau payangka is within the range reported for other populations of *Giuris* sp. The value of *b* (3.162) is slightly lower than that reported from Tondano Lake (Makmur *et al.* 2019) and Santani Lake in Papua and close to the lower limit of *Giuris* sp. from Limboto Lake. However, a value of *b* > 3 indicates that food availability is unlikely to be a limiting factor.

**Table 7.** Length-weight parameters and size range of *Giuris* sp. from sites in Indonesia.

Site	Sample		Length-weight parameters		Source
	n	Size range (cm)	*a*	*b*	
1. Bolano Sau Lake	107	7.9-16.3	0.0087	3.162	This study
2a. Tondano Lake (male)	249	10.7-19.0	0.0054	3.27	^10^
2b.Tondano Lake (female)	353	10.5-20.5	0.004	3.38	
3. Limboto Lake	309	no data	0.003-0.005	3.13-3.36	^108^
4. Sentani Lake, Papua	64	no data	0.0044	3.36	( http://www.fishbase.org)

The length distribution of *Giuris* spp. specimens in this study was biased towards fish large enough to have potentially achieved sexual maturity. This is important because all samples were collected with gears (throw nets and gillnets) currently used by the Bolano Sau fishing community. With respect to the gillnet fishery, 67% of fish caught during the baseline survey of Bolano Sau Lake were found to be sexually mature, with a mean size at first maturity (both sexes combined) of 11.92 cm. ^45^ Re-analysing the data disaggregated by sex ( Figure 5), the mean size at capture was greater than the estimated size at sexual maturity for both sexes. This analysis also indicates that the fishery is selective for males, and could explain the male bias previously reported for this population. ^45^

A study of *Giuris* spp. (most likely *G. aporocephalus*) in Tondano Lake, North Sulawesi ^10^ also found a larger mean length in males (14.15 cm) than in females (13.75 cm), although the difference was less marked than for the Bolano Sau Lake *payangka* identified as *G. laglaizei* in this study. Furthermore, mean and maximum sizes sampled for both sexes in Tondano Lake were larger than in Bolano Sau Lake ( Table 7). The generally smaller size of *payangka* in Bolano Sau Lake compared with Tondano Lake ^10^ could be related to inter-specific differences and/or environmental factors. A comparison between selected morphometric characters of the Bolano Sau Lake *payangka* and eight *Giuris* species recently described or re-described is presented in Table 8 while Table 9 shows comparative data on meristic characters.

**Table 8.** Comparison of selected morphometric characters within the genus *Giuris.*

No	Species ^a^	n	Nearest whole percentage of standard length (SL)				
			Head length	Body depth	CP ^b^ depth	Jaw length	Eye diameter
1	*Giuris laglaizei* ( *payangka*)	42	27-40	19-35	12-22 ^c^	6-11	4-7
2	*G. aporocephalus*	12	31-36	20-25	13-16	9-11	5-7
3	*G. caussei*	2	36	22-25	14-16	10	4-6
4	*G. charpini*	8	31-33	19-25	13-16	10-11	5-7
5	*G. laglaizei*	7	30-35	22-27	14-17	8-11	5-6
6	*G. margaritaceus*	12	30-35	20-25	13-16	10-11	6-8
7	*G. tolsoni*	11	31-37	20-24	13-15	9-12	6-7
8	*G. viator*	10	31-35	20-24	14-15	10-12	6-8
9	*G. yahayai*	10	31-36	26-39	16-20	9-12	4-6
10a	*G. margaritacea*	5 M	32	24	12	6	6
10b	*G. margaritacea*	5 F	32	25	13	6	5

^a^ Sources: No. 1: This study; No. 2-9: Ref. 15; No. 10: Ref. 10; Tondano Lake, 10a = males, 10b = females, mean values given.

^b^ CP = caudal peduncle; ^c^ One outlier each with 12% and 22%, remainder 15-20%.

**Table 9.** Comparison of meristic characters within the genus *Giuris.*

No	Species ^a^	n	Fin spines and rays ^b^					
			D1	D2	A	P	V	C
1	*Giuris laglaizei* ( *payangka*)	42	V-VI	I,7-8	I,8-10	12-15	I,4-5	11-17
2	*G. aporocephalus*	12	VI	I,8	I,9	14-15	I,5	13-14
3	*G. caussei*	2	VI	I,8-9	I,9	14-15	I,5	13
4	*G. charpini*	8	VI	I,8	I,8-9	13-14	I,5	13-14
5	*G. laglaizei*	7	VI	I,8	I,9	15	I,5	14-15
6	*G. margaritaceus*	12	VI	I,8	I,9	14-15	I,5	13-14
7	*G. tolsoni*	11	VI	I,8	I,9	14	I,5	13-15
8	*G. viator*	10	VI	I,8	I,9	14	I,5	13-14
9	*G. yahayai*	10	VI	I,8-9	I,9	14	I,5	15
10	*G. margaritacea* (5 male, 5 female)	10	VI	I,9	I,9	16	-	-
11	*G. margaritacea*	no data	VI	I,8	I,9	14-15	-	-

^a^ Sources: No. 1: This Study; No. 2-9: Ref. 53; No. 6-8: also Ref. 15; No. 10: Ref. 10; No. 11: http://www.fishbase.org.

^b^ D = dorsal fins (D1 = anterior; D2 = posterior); A = anal fin; P = pectoral fins; V = ventral (pelvic) fins; C = caudal fin.

Unlike the genetic (DNA barcoding) data, a comparison between morphometric and meristic characters of the Bolano Sau Lake payangka and the eight *Giuris* species recently described or re-described ^15^^,^^53^ in Tables 8 and 9 does not give a clear indication regarding the taxonomic identity of the Bolano Sau Lake *Giuris* population, although some characters are similar to the Philippine *Giuris laglaizei.* The wider range in Bolano Sau Lake *payangka* compared with most *Giuris* species for most of the characters could be related to the larger number of samples (42 compared with 2-12 fish). Although some morphometric characters have been used in discussing or determining the characteristics of species within the genus *Giuris*, in particular the resurrecting of synonymised species and the description of new species resulting in a total of eight now-recognised *Giuris* species, ^15^^,^^53^ there are many similarities. All eight are described as having a body shape which is more ovoid than elongated, with *G. yahayai* also having a somewhat backed appearance. Other common features include sexual dimorphism and known or suspected amphidromy.

### Life history of payangka (genus *Giuris*)

Amphidromy as described by McDowall (2007) is characterised by “reproduction in fresh water, passage to sea by newly hatched larvae, a period of feeding and growing at sea usually a few months long, return to fresh water of well-grown juveniles, a further period of feeding and growing in fresh water, followed by reproduction there” and can be obligate or facultative. ^89^ The presence of all life stages including adults (payangka) and larvae (nike) in Tondano Lake ^25^ indicates that amphidromy is most likely facultative in at least some *Giuris* species. Mean length at first maturity in Tondano Lake payangka has been reported as 10.75 cm (females) ^10^ and 13.4 cm (males)/13.7 cm (females). ^30^ Reported values for fecundity range from 12,000 to 127,000, increasing with female size. ^10^^,^^28^ Eggs fertilised in the morning hatch the following night and the larvae begin swimming after about 10 minutes, even though fins have not yet developed. ^28^ Although in the past all or some of these larvae may have been carried to the sea and completed an amphidromous life-cycle, the Tondano Lake *Giuris* population seems to have adopted a fully freshwater lifecycle. It has been proposed that, although this non-migratory lifestyle may have evolved within the population over a more extended time, it may have become the prevailing mode of reproduction as an adaptation to the construction of three hydroelectric power plant dams preventing downstream and upstream migrations. ^25^ Alternatively, if the Tondano Lake payangka was indeed introduced in 1902 from Limboto Lake, ^34^ then the adaptation may date from this introduction. The lack of genetic variation in the COI barcode for the six specimens sequenced by Pangemanan *et al.* ^25^ ( Figure 4) may be the result of a small founder population followed by such selection.

Whether amphidromy is obligate or facultative is an important consideration with respect to the Bolano Sau Lake payangka population. Despite the lake’s proximity to Tomini Bay, there is no permanent feature enabling fish to move between the lake and the sea. However, according to local fishermen seasonal flooding occurs at times during the raining season, creating a temporary connection. No larvae or small juveniles were found during the survey. While this may be an artefact of the collection methods used and/or the timing of the sampling, local fishermen do not catch nike in the lake, and did not report large schools of larvae. Further observations (monitoring) of Bolano Sau Lake payangka reproductive patterns and identification of early life stages in and/or near to the lake could shed light on this question.

If the payangka in Bolano Sau Lake is amphidromous and found in other waterbodies nearby, there is hope for natural recruitment from the wider Tomini Bay population to boost the population in the lake. The facilitation of such a process through the transport of migrating larvae (nike) is unlikely to be a wise move, as shoals of nike are typically multispecies, comprising several genera of Gobiidae and/or Eleotridae ^11^^,^^23^^,^^90^ as well as other taxa such as glass eels of the genus *Anguilla* and crustacea. ^12^^,^^19^^–^^21^ Therefore, shoals of *nike* are unlikely to be composed solely of *Giuris laglaizei* or indeed other species currently present in Bolano Sau Lake.

It is also possible that the payangka arrived in Bolano Sau Lake at some period(s) in the past when geological and hydrological features were more conducive to the migrations of diadromous fishes, and then adopted a fully freshwater life cycle, as seems to be the case for the payangka in Tondano Lake. In this case, it might be necessary to support recovery of the severely depleted *Giuris* population through well-planned release of captive bred fish. Initial steps towards captive breeding of the Tondano Lake payangka ( *Giuris* sp.) have been taken, including *ex-situ* husbandry of larvae and juveniles. ^28^ If indeed the *Giuris* sp. in Tondano Lake became established after the introduction of payangka from Limboto Lake in 1902, ^34^^,^^91^ it would indicate that such an approach might be successful. However, any such moves should follow the national guidelines for re-stocking, ^92^ in particular with respect to biosecurity (e.g. pests and disease), as well as ensuring the fish used for re-stocking are indeed the same species and ideally come from populations with similar genetic and other characteristics.

From an ecological perspective, trophic relations are an important consideration. Species reported as preying on *G. margaritacea* and/or *O. porocephala* include the piscivorous goby *Glossogobius giuris.* ^93^ The payangka in Tondano Lake is omnivorous but appears to undergo an ontogenetic shift in dietary preference, becoming increasingly carnivorous. Juveniles in the 12-30 mm TL range are reported as planktivorous, with the proportion of zooplankton relative to phytoplankton increasing as the fish grow; they begin to prey on *Caradina* shrimp at around 30 mm TL and on molluscs (Gastropods) and fish (including smaller conspecifics) at around 36-40 mm TL. ^31^ However, such data are lacking for the payangka from Bolano Sau Lake, and indeed for the species *G. laglaizei* in the Philippines.

### Conservation status and fisheries management

The IUCN Red List of Threatened Species re-assessment of the snakehead gudgeon *G. margaritacea* in 2019 ^76^ lists the species under the Least Concern (LC) category, with the rationale that the species is widespread and common in parts of its range, and that it is found in a wide variety of habitats. With respect to fisheries, the assessment mentions that this taxon is harvested for the international aquarium trade and in localised areas is also used for subsistence level consumption and as a bait fish, but levels of exploitation are unknown. The assessment also notes the need for further taxonomic work to determine if the Western Pacific and Western Indian Ocean subpopulations are conspecific. Recent genetic evidence validates this concern as the newly described *Giuris yahayai* appears to be limited to the Indian Ocean, ^53^ while seven species have been identified in the Indo-West Pacific with at least three species present in eastern Central Sulawesi, the Moluccas, and the Philippines. ^15^^,^^53^ The boundaries of each species cannot as yet be determined with certainty; however, the known ranges of several species vary in extent and in some cases overlap. ^53^ Together with the identification of the payangka from Bolano Sau Lake as *G. laglaizei*, the reanalysis of *Giuris* sequences from Tondano Lake *in* Pangemanan *et al.*, (2020) as belonging to a different clade from all other Sulawesian *Giuris* sequences (most likely *G. aporocephalus*) means that at least five *Giuris* species are present in Sulawesi, with four species found in Tomini Bay watersheds. Future barcoding studies may further increase the known range of one or several *Giuris* species.

The deep divisions in the genus *Giuris* call into question the validity of the LC status ^76^ for at least some of the species formerly considered as a single taxon, *G. margaritacea*. The fragmented nature and uneven size of known distributions indicate that some of the eight species currently apparent within this genus could be at risk from serial extirpation and even extinction. At least one *Giuris* population (species unknown) from the Proserpine River in Australia appears to have been lost. ^76^^,^^85^ It may never be known if this was a now extinct species or an extirpated population of a widespread species.

The principal threats mentioned in the Red List assessment ^76^ are subsistence and ornamental fisheries. However, alien or exotic invasive species introductions are considered a major threat to freshwater fish biodiversity worldwide. ^94^ In particular, wild Nile tilapia ( *Oreochromis niloticus*) populations are increasingly widespread across Indonesia ^39^^–^^42^^,^^95^^–^^97^ and have been implicated in the decline of native species in many inland waters across the Indonesian Archipelago. ^39^^,^^40^^,^^97^^–^^99^ Mechanisms through which introduced species, including the Nile tilapia, could affect the payangka and other native fishes include competition for food and habitat; the introduction and transmission of parasites and disease; predation, especially on eggs and larvae or juveniles; and behaviour leading to habitat degradation ^39^^,^^41^^,^^97^^,^^100^^–^^102^

The majority of data on *Giuris* fisheries are from Tondano Lake in North Sulawesi, approximately 500 km east of Bolano Sau Lake. Payangka has historically been the main fisheries species in Tondano Lake, comprising 35% of the total production volume in 1980. ^28^^,^^100^ All sizes from 9 mm to 200 mm are reported in fisheries catch ^28^^,^^31^; however the nike fisheries target postlarvae and fingerlings in the 12-30 mm TL range, while payangka catches were dominated by the size range 105-135 mm TL, with few larger individuals. While the nike fishery is economically viable, with relatively high income and profit margins, ^27^ concerns have been expressed regarding the aggregate ecological sustainability of the fisheries targeting *payangka* in the lake. ^30^ Strong indications of overfishing were already apparent around 30 years ago, with annual catch volume reduced to 25% of that in 1980-1985 by 1990. ^31^ The introduction of alien species has been implicated as a causal factor of declining *Giuris* catches and abundance. In North Sulawesi, introduced catfish ( *Clarias* sp.) are reported to have had negative impacts on payangka stocks in Tondano Lake. ^100^ However, it seems the payangka itself may be an introduced species in this waterbody. ^34^

With respect to the Bolano Sau Lake payangka, this once common and popular food fish has become increasingly rare since the introduction of alien species under government programs intended to increase fisheries production, although overfishing is also suspected as a factor. ^45^ In Bolano Sau Lake, government-supported “re-stocking” has occurred in the lake over several decades. ^35^ The most abundant alien species in 2019 was the Nile tilapia ( *Oreochromis niloticus*), while other introduced alien species included the Mozambique tilapia ( *Oreochromis mossambicus*) and gourami ( *Trichogaster* sp.). Two other species, the striped snakehead *Channa striata* and climbing perch *Anabas testudineus*, are widely considered as native fishes by Sulawesians and figure in many traditional dishes, although they may have been introduced to Sulawesi, possibly in prehistoric times. ^6^^,^^103^^,^^104^ With respect to introduced species, it is interesting to note that not all species introduced to Bolano Sau Lake have become established or invasive. For example, the common carp ( *Cyprinus carpio*) had been repeatedly introduced prior to 2016, ^35^ but none were seen during the surveys in 2019. Fishermen reported that after the introduction they did catch carp; but the numbers dwindled over time, in contrast to the Nile tilapia ( *O. niloticus*) which quickly became the dominant species in the lake.

The IUCN Red List assessment ^76^ describes the habitat of *G. margaritaceus* as streams, while in Northern Australia the most common habitat is described as small rainforest creeks and wetlands located close to the river mouth ^85^; lacustrine habitat is not mentioned. However, *Giuris* species have been found in both coastal streams and lakes, and would seem that lakes are a key habitat for at least some *Giuris* species in Indonesia, and specifically in Sulawesi, as well as in the Philippines and Papua New Guinea. ^15^^,^^52^^,^^53^^,^^105^ It would seem likely that *G. laglaizei* may be a predominantly lacustrine species, as the known Philippine populations are all lacustrine, as is the Sulawesi population in this study.

As in many regions worldwide, ^94^ lakes in this region are typically subject to significant anthropogenic disturbance leading to habitat alteration and degradation. ^40^^,^^41^^,^^106^^,^^107^ This includes lakes known to have *Giuris* populations such as Limboto and Tondano, ^100^^,^^108^ with negative impacts on payangka stocks including changes in condition factor. ^100^ Quantitative data ( Table 5) and qualitative observations indicate potential threats to the Bolano Sau Lake environment as a habitat for fish. Parameters of concern include temperature, consistently above 31°C with a maximum in excess of 33°C, and dissolved oxygen (DO). The latter was consistently below 4 mg/L and sometimes below 3 mg/L, the lower limit considered acceptable by Government Regulation No. 82/2001. ^109^ The low levels of DO may be related to the elevated temperature, as the capacity to retain oxygen is inversely correlated with water temperature. ^110^ The gill oxygen limitation theory (GOLT) proposed by Pauly ^111^ posits that fish growth and size are limited by the availability of oxygen; higher temperatures increase metabolic rates, lowering the size at which the limitation will be reached. Thus, the high temperature and low DO values recorded in Bolano Sau Lake could be a contributing factor to the relatively small maximum size of the payangka. Locally high levels of nitrate (NO3-N) may be due to sewage and/or fertiliser run-off, and could and act in synergy with the temperature and low oxygen conditions, especially as toxicity increases with temperature. ^110^ Combined with the observed land use/land cover changes, these data call for integrated watershed management.

## Conclusion

The COI barcoding approach identified a fish locally called payangka from Bolano Sau Lake in Central Sulawesi Indonesia as the recently resurrected *Giuris laglaizei.* This represents the first record of *G. laglaizei* in Indonesia and indeed the first outside the Philippines. However, in contrast to the molecular approach, the phenotypic characters measured or counted in this study could not enable a definitive identification of the *Giuris* sp. in Bolano Sau Lake to species level. In addition, other characteristics noted during this study and other visits to the study site are ambiguous in terms of taxonomic identification. For example, both colour and general appearance vary between sexes, stage of the reproductive cycle, and even habitat characteristics within the lake. These results and considerations strongly indicate the advisability of further research using molecular biology methods to resolve the taxonomic identity of other *Giuris* sp. populations throughout the distribution of this genus, including the use of multiple molecular markers.

The phylogenetic analysis of *Giuris* highlights the complex biogeography of this genus in Indonesia, with at least four *Giuris* species present in the coastal regions around Tomini Bay and five in Sulawesi. These findings call into question the IUCN Red List Least Concern status of the taxonomic unit, until recently named *Giuris margaritacea,* now revealed as a genus comprising at least eight species. While identifying the species present is a first step towards managing and preserving fish biodiversity in inland waters, this needs to be followed by appropriate management. The threats to and sharp decline of the Bolano Sau Lake payangka population are reflected in similar threats and/or trends reported for other *Giuris* spp. populations, and could result in serial extirpations if unchecked. From both biodiversity and fisheries perspectives, there is a need to manage freshwater fisheries resources to conserve native fish species still present, including the Bolano Sau Lake payangka.

## Data availability

### Underlying data

Harvard Dataverse: Bolano Sau Lake Fish Data https://doi.org/10.7910/DVN/JL5JP9. ^69^

This project contains the following underlying data:

• Giuris_payangka_data_2019.tab (dataset)

Data are available under the terms of the Creative Commons Zero “No rights reserved” data waiver (CC0 1.0 Public domain dedication).

NCBI Metazoan Mitochondrial COX1 SUB10960949 ^63^:

• The partial Cytochrome C Oxidase subunit I gene mitochondrial DNA sequence of *Giuris laglaizei*, Accession OM674613 https://www.ncbi.nlm.nih.gov/nuccore/OM674613
• The partial Cytochrome C Oxidase subunit I gene mitochondrial DNA sequence of *Anabas testudineus*, Accession OM674614 https://www.ncbi.nlm.nih.gov/nuccore/OM674614

### Extended data

Harvard Dataverse: GenBank Accessions, BOLD Records (mitochondrial COI gene sequences) and other nucleotide sequences used for phylogenetic analyses of Eleotridae and *Giuris* spp. https://doi.org/10.7910/DVN/WMDHOJ. ^112^

This project contains the following extended data:

• Table S1_mtDNA_COI_sequence_references.pdf (Table S1)

Data are available under the terms of the Creative Commons Zero “No rights reserved” data waiver (CC0 1.0 Public domain dedication).

### Reporting guidelines

Harvard Dataverse: ARRIVE checklist for ‘DNA barcoding detects resurrected taxon Giuris laglaizei (Sauvage 1880) in Sulawesi, Indonesia: Bolano Sau Lake payangka phylogeny, phenotypic characters and implications for Giuris spp. conservation’ https://doi.org/10.7910/DVN/JL5JP9. ^69^

Data are available under the terms of the Creative Commons Zero “No rights reserved” data waiver (CC0 1.0 Public domain dedication).
