# Supplementary material for: DNA barcoding detects resurrected taxon Giuris laglaizei (Sauvage 1880) in Sulawesi, Indonesia: Bolano Sau Lake payangka phylogeny, phenotypic characters and implications for Giuris spp. conservation
Source: F1000Res. 2023 Jan 26;11:295. Originally published 2022 Mar 10. [Version 2] doi: 10.12688/f1000research.108970.2 (PMC9936567; doi:10.12688/f1000research.108970.2)
Supplement: Peer review history

# Peer review: Reviewer response for version 2

**DOI:** 10.5256/f1000research.143553.r161563

**Published:** 2023-02-16

**Authors:** Jonas P. Quilang

**Affiliations:** 1 Institute of Biology, University of the Philippines Diliman, Quezon City, Philippines

I don't have further comments on the revised article.

Is the work clearly and accurately presented and does it cite the current literature?

Yes

If applicable, is the statistical analysis and its interpretation appropriate?

Yes

Are all the source data underlying the results available to ensure full reproducibility?

Yes

Is the study design appropriate and is the work technically sound?

Partly

Are the conclusions drawn adequately supported by the results?

Yes

Are sufficient details of methods and analysis provided to allow replication by others?

Yes

Reviewer Expertise:

NA

I confirm that I have read this submission and believe that I have an appropriate level of expertise to confirm that it is of an acceptable scientific standard.

# Peer review: Reviewer response for version 2

**DOI:** 10.5256/f1000research.143553.r161562

**Published:** 2023-01-27

**Authors:** Aurycéia Guimarães-Costa

**Affiliations:** 1 Institute of Coastal Studies, Universidade Federal do Pará, Bragança, Brazil

After reviewing the corrections made by the authors, I consider them all to be very appropriate. With no further considerations to make, I approve for publication.

Is the work clearly and accurately presented and does it cite the current literature?

Yes

If applicable, is the statistical analysis and its interpretation appropriate?

Yes

Are all the source data underlying the results available to ensure full reproducibility?

Yes

Is the study design appropriate and is the work technically sound?

Yes

Are the conclusions drawn adequately supported by the results?

Yes

Are sufficient details of methods and analysis provided to allow replication by others?

Yes

Reviewer Expertise:

Evolution, DNA Barcoding, phylogeny

I confirm that I have read this submission and believe that I have an appropriate level of expertise to confirm that it is of an acceptable scientific standard.

# Peer review: Reviewer response for version 1

**DOI:** 10.5256/f1000research.120417.r137863

**Published:** 2022-07-18

**Authors:** Aurycéia Guimarães-Costa

**Affiliations:** 1 Institute of Coastal Studies, Universidade Federal do Pará, Bragança, Brazil

The manuscript by Ndobe *et al.* brings up taxonomic information about a group of species that have many difficulties in morphological identification, which are members of the Eleotridae family. The authors presented a brief history of the revisions of the taxonomic nomenclatures that the species of Giuris passed, showing that there are still confusions in the delimitation of the species.

 In the work, the authors used the DNA Barcoding methodology to show that specimens regularly identified as *G. margaritacea*/ *G. margaritaceus* are actually Giuris laglaizei, in addition to identifying the first occurrence of this species in Indonesia.

 Overall, I liked the manuscript. The objectives were achieved based on the DNA Barcoding methodology. Although this methodology is very direct and objective, the authors also elucidated other questions related to the taxonomy of the group, such as, for example, the elucidation of a species complex ( *Giuris margaritacea*) that may comprise at least eight species. This evidence is extremely important for the delimitation of conservation care for the correct species.

 Thus, this study, which integrated morphology and molecular, needs to be disseminated to science and, mainly, to researchers interested in the evolutionary relationships of the Eleotridae family.

 Minor revisions

- Background: change “seventeen" to “17”
- I suggest changing all mentions of the name "Payangka" that are in italic font to regular font, as even mentioned in the text as a popular name for the species, an italicized name can be confused with the genus.
- I think the tree in Figure 4 is not didactic for visualizing Giuris' clusters. I strongly advise rendering the tree to a linear topology, so that the reader does not have a hard time seeing the formation of the groups and the names of the species.

Is the work clearly and accurately presented and does it cite the current literature?

Yes

If applicable, is the statistical analysis and its interpretation appropriate?

Yes

Are all the source data underlying the results available to ensure full reproducibility?

Yes

Is the study design appropriate and is the work technically sound?

Yes

Are the conclusions drawn adequately supported by the results?

Yes

Are sufficient details of methods and analysis provided to allow replication by others?

Yes

Reviewer Expertise:

Evolution, DNA Barcoding, phylogeny

I confirm that I have read this submission and believe that I have an appropriate level of expertise to confirm that it is of an acceptable scientific standard.

# Response

**Published:** 2023-01-23

**Authors:** Samliok Ndobe

**Affiliations:** Universitas Tadulako, Indonesia

Thank you very much for your input and approval. Please see below detailed response to your suggestions for **Minor revisions**

 1. Background: change “seventeen" to “17”

**Response: **Thank you, we have made this change.

 2. I suggest changing all mentions of the name "Payangka" that are in italic font to regular font, as even mentioned in the text as a popular name for the species, an italicized name can be confused with the genus.

**Response: **Thank you, we have made this change.

 3. I think the tree in Figure 4 is not didactic for visualizing Giuris' clusters. I strongly advise rendering the tree to a linear topology, so that the reader does not have a hard time seeing the formation of the groups and the names of the species.

**Response: **Thank you for the suggestion, but we have not made this suggested change. Using a linear topology makes the tree too long to fit (at a readable size) on a standard page or on a laptop screen. We could produce the tree in a linear format and add it to the "Extended Data" deposited in the on-line repository. Please advise if you think that would be a good idea.

# Peer review: Reviewer response for version 1

**DOI:** 10.5256/f1000research.120417.r135393

**Published:** 2022-05-23

**Authors:** Jonas P. Quilang

**Affiliations:** 1 Institute of Biology, University of the Philippines Diliman, Quezon City, Philippines

The authors collected *Payangka* specimens from August to December 2019 in Bolano Sau Lake in Parigi Moutong District, Central Sulawesi Province, Indonesia. They recorded the length, weight and sex of 111 specimens and obtained data for 17 morphometric and six meristic characters from 42 specimens. These morphometric and meristic data were used for morphological analysis and determination of length-weight relationship. In addition, the authors obtained the mitochondrial cytochrome c oxidase I (COI) sequence from a single specimen for molecular identification via DNA barcoding. Additional COI sequences were obtained from public databases for phylogenetic analysis. DNA barcoding revealed that the specimen matched with *Giuris laglaizei* in public databases.

 The study is interesting because this is the first record for this species outside of its known distribution (Philippines). However, it could have been better if additional specimens were sequenced for their COI so as to have replication and also to be able to compute for intra-species genetic distance and to determine if there is genetic differentiation within the species. COI sequencing of additional specimens may reveal not only genetic differentiation but also presence of cryptic species.

 COI is an established marker in fishes and other animals for DNA barcoding, that is, for species identification and delineation. However, for phylogenetic studies additional molecular mitochondrial and nuclear markers have to be used and so caution should be observed when using the terms phylogenetic and polyphyletic in the manuscript. A more comprehensive phylogenetic analysis which should include all species under the genus *Giuris* and other genera under Eleotridae and using not just a single molecular marker (COI) should be done before one can conclude that *Giuris* is indeed polyphyletic and not monophyletic or paraphyletic; hence, the authors should avoid using the term polyphyletic.

 Lastly, the colon in the title is usually used to separate the main part (which is broader in scope) from the subtitle or explanatory part. It seems that the use of the colon in the title is inappropriate. The first part of the title is declarative while the second part (to the right of the colon, which is supposedly the subtopic), is descriptive and so there is a disconnect. I suggest that the title be made more specific and more reflective of what was done in the study. The bulk of the study is on morphological analysis, hence, this should be given more emphasis in the title. Only a single DNA sequence was generated in this study, which was used for the DNA barcoding and phylogenetics aspects of the study.

 Please see additional comments and corrections in the attached copy of the manuscript.

Is the work clearly and accurately presented and does it cite the current literature?

Yes

If applicable, is the statistical analysis and its interpretation appropriate?

Yes

Are all the source data underlying the results available to ensure full reproducibility?

Yes

Is the study design appropriate and is the work technically sound?

Partly

Are the conclusions drawn adequately supported by the results?

Yes

Are sufficient details of methods and analysis provided to allow replication by others?

Yes

Reviewer Expertise:

Population genetics, molecular genetics and phylogenetics, fish biology

I confirm that I have read this submission and believe that I have an appropriate level of expertise to confirm that it is of an acceptable scientific standard, however I have significant reservations, as outlined above.

# Response

**Published:** 2023-01-23

**Authors:** Samliok Ndobe

**Affiliations:** Universitas Tadulako, Indonesia

Thank you very much for your thoughful input. Dey=tailed responses are given below.

 The study is interesting because this is the first record for this species outside of its known distribution (Philippines). However, it could have been better if additional specimens were sequenced for their COI so as to have replication and also to be able to compute for intra-species genetic distance and to determine if there is genetic differentiation within the species. COI sequencing of additional specimens may reveal not only genetic differentiation but also presence of cryptic species.

**Response: **we also consider that this additional work would be worthwhile. We are exploring the possibility of a more detailed study on payangka (possibly focused on more than one lake) in order to address such additional research questions, which were not within the scope of the research reported here.

 COI is an established marker in fishes and other animals for DNA barcoding, that is, for species identification and delineation. However, for phylogenetic studies additional molecular mitochondrial and nuclear markers have to be used and so caution should be observed when using the terms phylogenetic and polyphyletic in the manuscript. A more comprehensive phylogenetic analysis which should include all species under the genus Giuris and other genera under Eleotridae and using not just a single molecular marker (COI) should be done before one can conclude that Giuris is indeed polyphyletic and not monophyletic or paraphyletic; hence, the authors should avoid using the term polyphyletic.

**Response:** thank you for this advice. We accept the point you are making and have removed all occurrences of the term polyphyletic, using expressions which we consider reflect the results we can consider as robust. With respect to the term phylogenetic, we consider that we have used this term sparingly, in two ways. Firstly, as a description of what we did (construction of phylogenetic trees), and secondly referring to the interpretation (phylogenetic analysis) of results from both our study (in particular the phylogenetic trees) and other studies. With respect to other markers, again that is outside the scope of this study. However, we fully agree that further research is needed, and indeed the need for such research is one of our conclusions. In the revised version we have explicitly stated the need for additional molecular markers in the conclusion.

 Lastly, the colon in the title is usually used to separate the main part (which is broader in scope) from the subtitle or explanatory part. It seems that the use of the colon in the title is inappropriate. The first part of the title is declarative while the second part (to the right of the colon, which is supposedly the subtopic), is descriptive and so there is a disconnect. I suggest that the title be made more specific and more reflective of what was done in the study. The bulk of the study is on morphological analysis, hence, this should be given more emphasis in the title. Only a single DNA sequence was generated in this study, which was used for the DNA barcoding and phylogenetics aspects of the study.

**Response: **thank you for this input, which we will certainly consider for the future. We have not edited the title in this revised version because of the publishing model of F1000 Research; as the paper was published on-line before review, we consider this could cause confusion. Additionally, the Editor and Reviewer 2 have not requested a change of title.

 Please see additional comments and corrections in the attached copy of the manuscript.

**Response: **Thank you, we have addressed these points in the revised manuscript. We also made an annotated version of the pdf copy you kindly supplied, giving more detail on our edits in response to your suggestions and comments. We will endeavor to share this file if it is possible.
